# Retroperitoneal Leiomyosarcoma Presenting as Lower Gastrointestinal Bleeding: A Case Report and Review of the Literature

**DOI:** 10.1155/2011/358680

**Published:** 2011-09-08

**Authors:** Dominic G. Ventura, Shyam J. Thakkar, Katie Farah

**Affiliations:** Allegheny Center for Digestive Health, West Penn Allegheny Health System, Pittsburgh, PA 15212, USA

## Abstract

We report the first known case of a retroperitoneal leiomyosarcoma that presented with an endoscopically defined source of gastrointestinal bleeding in the colon. A 68-year-old male with a history of diverticulosis, hypertension, and hypercholesterolemia who complained of a 3-month history of abdominal pain, nausea, and intermittent hematochezia presented for evaluation of large volume hematochezia and lightheadedness. Colonoscopy revealed left-sided diverticulosis and rectal varices without stigmata of recent bleed. CT scan showed a 26 × 20 × 13 cm heterogeneous retroperitoneal mass and multiple hypodense hepatic lesions. Liver biopsy revealed leiomyosarcoma. In summary, although surgery is the mainstay of treatment, resectability has not improved significantly. Early recognition and aggressive surgery are keys to long-term survival.

## 1. Introduction

Retroperitoneal soft-tissue sarcomas rare. They are locally invasive, large tumors that usually present with abdominal discomfort and palpable mass. They rarely present with lower gastrointestinal bleeding. We report the first known case of a retroperitoneal leiomyosarcoma that presented with an endoscopically defined source of gastrointestinal bleeding in the colon.

## 2. Case Presentation

We present a case of a 68-year-old male with a history of diverticulosis, hypertension, and hypercholesterolemia who complained of a 3-month history of abdominal pain, nausea, and intermittent hematochezia. He presented to the emergency room for evaluation of large volume hematochezia and lightheadedness. 

Physical examination revealed a systolic blood pressure of 80 mmHg. Abdominal exam showed mild distension and diffuse tenderness. Nasogastric lavage was clear. Rectal exam was notable for bright red blood, but no mass or hemorrhoids. Laboratory evaluation showed blood urea nitrogen (BUN) = 35 mg/dL, creatinine = 1.9 mg/dL, international normalized ratio (INR) = 1.4, hemoglobin = 4.8 g/dL, and platelet count = 498,000 k/mcl. 

**Figure 1:**
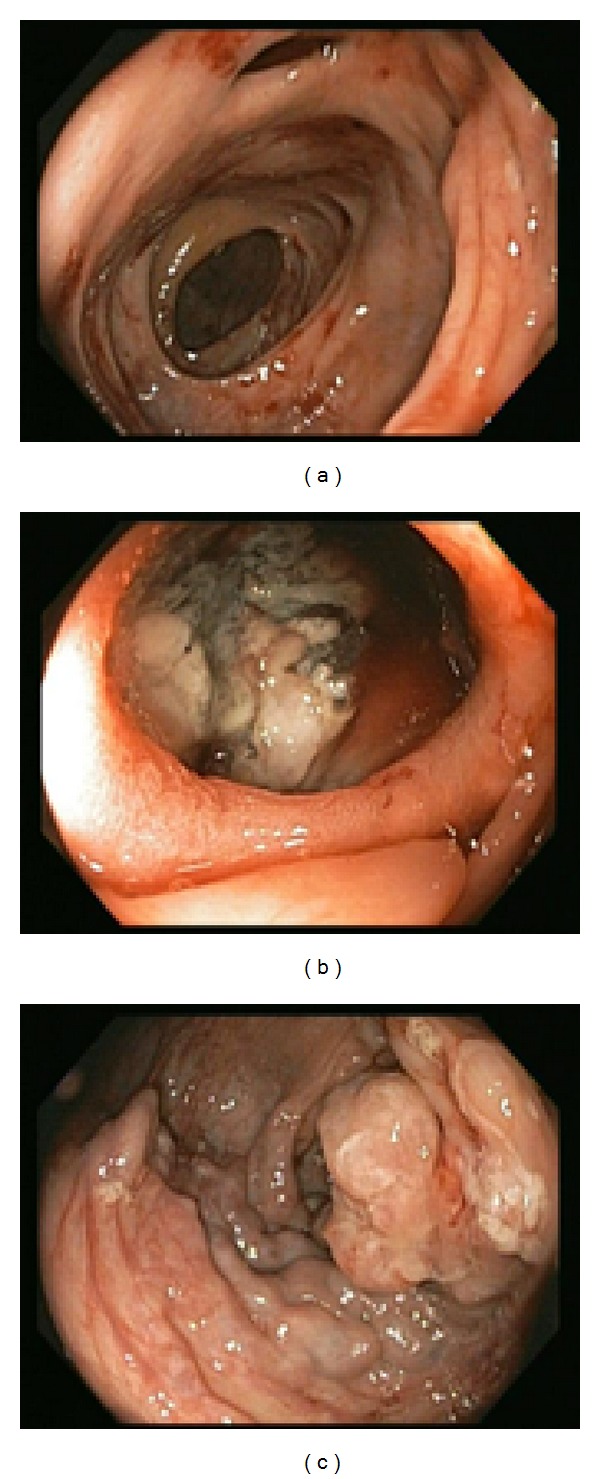
(a) Ascending colon with ileocecal valve in the distance and cavity at the 12 o'clock position. (b) View into cavity. (c) Mass and varices seen distal to the cavity.

**Figure 2:**
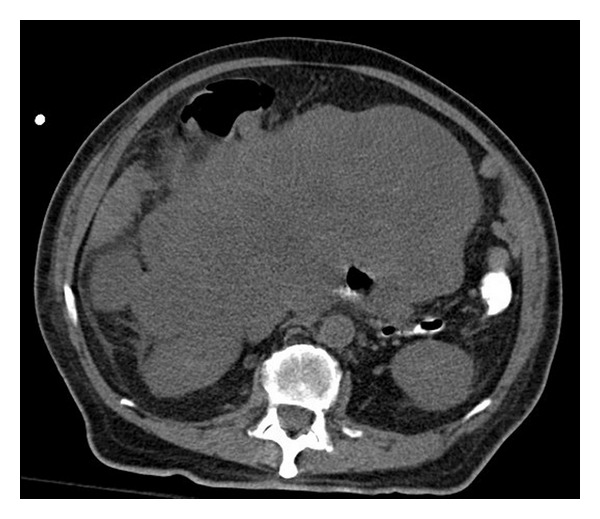
Large retroperitoneal mass near right colon and SMA.

Upper endoscopy revealed a hiatal hernia and extrinsic compression of the second part of the duodenum, but there were no signs of active or recent bleeding. Colonoscopy revealed left-sided diverticulosis and rectal varices without stigmata of recent bleed. Additionally, a large cavity was found in the proximal ascending colon with a necrotic mass and surrounding varices (Figure 1). No active bleeding was observed. CT scan showed a 26 × 20 × 13 cm heterogeneous retroperitoneal mass, multiple hypodense hepatic lesions, grade 2 right-sided hydronephrosis, and mildly dilated proximal small bowel with no evidence of small bowel obstruction (Figure 2). Liver biopsy revealed leiomyosarcoma.

The patient underwent exploratory laparotomy for palliative right hemicolectomy and was found to have a large tumor extending from the right retroperitoneum lateral to the right colon and anterior to the right kidney. The mass was invading the colonic mesentery and encased the proximal one-third of the superior mesenteric artery. Therefore, he was deemed inoperable and was discharged home with hospice care.

## 3. Discussion

Soft-tissue sarcomas are rare accounting for approximately 1% of adult malignancies. Fifty percent of these occur in the extremities followed by retroperitoneal/visceral and trunk involvement [1]. Retroperitoneal soft-tissue sarcomas account for 13% of all adult soft-tissue sarcomas with malignant fibrous histiocytomas and liposarcomas being the most common. They are locally invasive, large tumors that remain occult for long periods of time due to the abdominal cavity's ability to accommodate these slowly expanding masses with a paucity of symptoms [2]. Most cases present with abdominal discomfort (60%–70%) and palpable mass (70%–80%) but rarely do they present with lower gastrointestinal bleeding [3]. 

There have been 36 cases reported of duodenocaval fistula and one case of gastric invasion of retroperitoneal sarcomas causing gastrointestinal bleeding [4, 5], all of which have been after surgical resection and/or radiation therapy. One case has been reported of a lower gastrointestinal bleed from direct invasion of a retroperitoneal liposarcoma invading the terminal ileum; however, the site could not be found endoscopically [6]. This is the first known case of a retroperitoneal leiomyosarcoma that presented with an endoscopically defined source of gastrointestinal bleeding in the colon. 

The overall 5-year survival rate of retroperitoneal sarcomas 36%–58% and is dependent on tumor histology and extent of tumor invasion [7]. Surgery is the mainstay of treatment, but despite improved imaging techniques and preoperative and intraoperative patient management, the incidence of successful resection has not improved significantly in the past 20 years. Even with aggressive surgical techniques, only one half of tumors can be resected completely, and of those, up to 90% result in local recurrence and result in death [8]. In a case series of 73 patients treated from 1984 to 2003, 69.8% were resected completely with a local recurrence rate of 37.2% and a 5-year survival of 58%. The 5-year survival of the remaining patients was 0% [9].

In contrast to the promising results seen with extremity soft-tissue sarcoma and childhood sarcomas, the addition of adjuvant radiotherapy or chemotherapy has not altered prognosis. In a review of 312 patients with abdominal and truncal sarcomas treated from 1977 to 2004, the ability to resect the lesion, negative resection margins, and tumor histology were the only good prognostic factors [10]. Liposarcomas and fibrosarcomas had better outcomes than leiomyosarcomas or malignant fibrous histiocytomas. Chemoradiotherapy did not improve outcomes. Adjuvant and neoadjuvant chemoradiotherapy are being used but further investigation is necessary [11]. 

Early recognition and aggressive surgery are the keys to long-term survival of patients with retroperitoneal sarcomas, but the mainstay of treating advanced disease is yet to be determined and needs further research as the overall prognosis of advanced disease is relatively poor.

## References

[1] Hoos A, Lewis JJ, Brennan MF (2000). Soft-tissue sarcomas—prognostic factors and multimodal therapy. *Chirurg*.

[2] Lawrence W, Donegan WL, Natarajan N (1987). Adult soft tissue sarcomas : a pattern of care survey of the American College of Surgeons. *Annals of Surgery*.

[3] Wanchick K, Lucha P (2009). Dedifferentiated retroperitoneal liposarcoma presenting as lower gastrointestinal bleeding, a report and review of the literature. *Military Medicine*.

[4] Perera GB, Wilson SE, Barie PS, Butler JA (2004). Duodenocaval fistula: a late complication of retroperitoneal irradiation and vena cava replacement. *Annals of Vascular Surgery*.

[5] Moriya K, Masui K, Maekawa Y (2005). Retroperitoneal leiomyosarcoma and leiomyoblastoma directly invaded the stomach: an autopsy case report. *Journal of Nara Medical Association*.

[6] Wanchick K, Lucha P (2009). Dedifferentiated retroperitoneal liposarcoma presenting as lower gastrointestinal bleeding, a report and review of the literature. *Military Medicine*.

[7] Porter GA, Baxter NN, Pisters PW (2006). Retroperitoneal sarcoma: a population-based analysis of epidemiology, surgery, and radiotherapy. *Cancer*.

[8] Storm FK, Mahvi DM (1991). Diagnosis and management of retroperitoneal soft-tissue sarcoma. *Annals of Surgery*.

[9] Pacelli F, Tortorelli AP, Rosa F (2008). Retroperitoneal soft tissue sarcoma: prognostic factors and therapeutic approaches. *Tumori*.

[10] Perez EA, Gutierrez JC, Moffat FL (2007). Retroperitoneal and truncal sarcomas: prognosis depends upon type not location. *Annals of Surgical Oncology*.

[11] Thomas DM, O'Sullivan B, Gronchi A (2009). Current concepts and future perspectives in retroperitoneal soft-tissue sarcoma management. *Expert Review of Anticancer Therapy*.

